# Supplementary material for: Driver gene combinations dictate cutaneous squamous cell carcinoma disease continuum progression
Source: Nat Commun. 2023 Aug 25;14:5211. doi: 10.1038/s41467-023-40822-9 (PMC10457401; doi:10.1038/s41467-023-40822-9)
Supplement: Supplementary file 1 — Supplementary Information [file 41467_2023_40822_MOESM1_ESM.pdf]

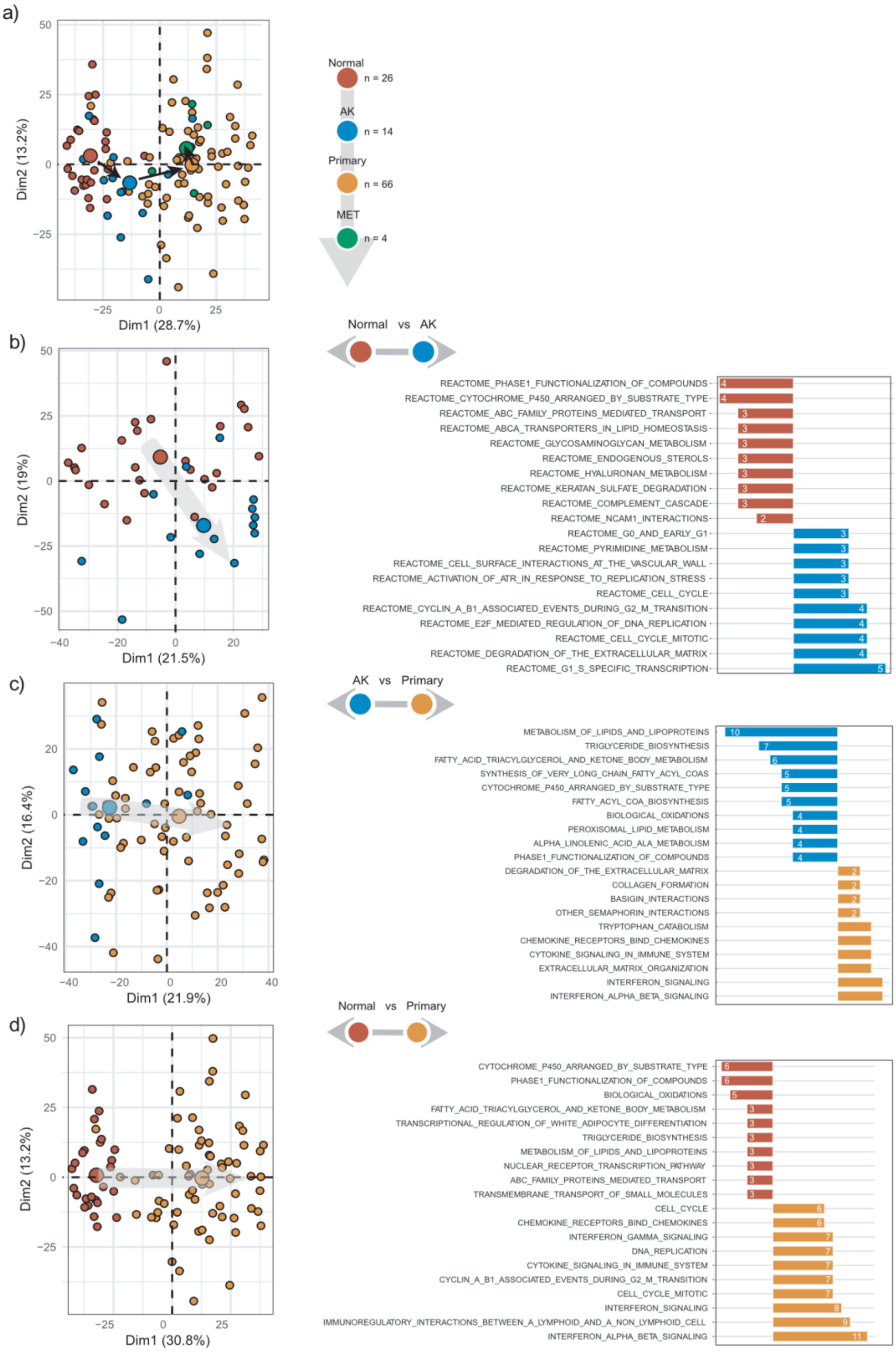

Supplementary Figure 1. Principal Component Analysis (PCA) analysis and gene set enrichment analysis of 110 samples from normal skin, AK, primary and metastatic cSCC samples identifies pathways and processes that alter between disease states. a) PCA of the top 2000 most variably expressed genes showing clustering of samples according to clinical designation; normal skin (red), AK (blue), primary (orange) and metastatic cSCC (MET, green). Mean points of each clinical designation are presented as larger coloured circles joined by black arrows which indicate the direction of disease progression. **b)** PCA of the top 2000 most variably expressed genes

expressed in normal skin and AK samples. Mean points of the respective clinical designations are presented as larger coloured circles. The grey arrow indicates the direction of disease progression. Gene set enrichment analysis showing molecular pathways and/or processes significantly enriched in normal skin (red bars) or AK samples (blue bars). -Log10(Pvalues) are presented in each bar. **c)** PCA of the top 2000 most variably expressed genes in AK and primary cSCC samples. Mean points of the respective clinical designations are presented as larger coloured circles. The grey arrow indicates the direction of disease progression. Gene set enrichment analysis showing molecular pathways and/or processes significantly enriched in AK (blue bars) or primary cSCC samples (orange bars). -Log10(Pvalues) are presented in each bar. **d)** PCA of the top 2000 most variably expressed genes in normal skin and primary cSCC samples. Mean points of the respective clinical designations are presented as larger coloured circles. The grey arrow indicates the direction of disease progression. Gene set enrichment analysis showing molecular pathways and/or processes significantly enriched in normal skin (red bars) or primary cSCC samples (orange bars). -Log10(Pvalues) are presented in each bar.

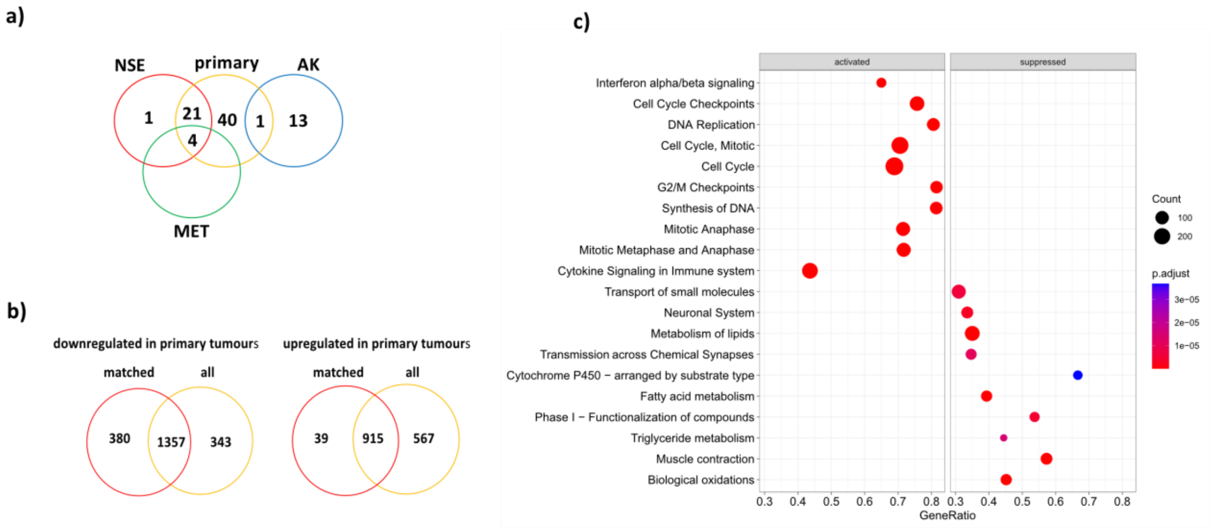

Supplementary Figure 2. Matched sample analysis reveals pathways and processes altered in primary tumours compared to normal sun exposed skin. **a)** Venn diagram indicating the relationship of human samples with overlaps indicating samples that are derived from the same patient (NSE, normal sun exposed skin, AK, actinic keratosis, MET, metastasis). **b)** Venn diagram indicating the number of genes downregulated and upregulated (padj<0.05, log2 FC>1) in primary tumours compared to normal skin samples in both the matched pair analysis (matched) from 25 matched normal skin and primary tumour samples and the analysis when comparing all normal skin (n=26) and all primary tumour samples ((n=66, (all)). **c**) Gene set enrichment analysis of reactome pathways from the matched sample analysis showing the top 10 pathways downregulated (suppressed) or upregulated (activated) in primary tumours compared to normal skin samples. Dots coloured by one-sided p-values from Fisher's test (labelled as p.adjust) by the clusterProfiler R package. P values <= 0.05 indicating a significant difference between sample groups. P-values not adjusted for multiple testing.

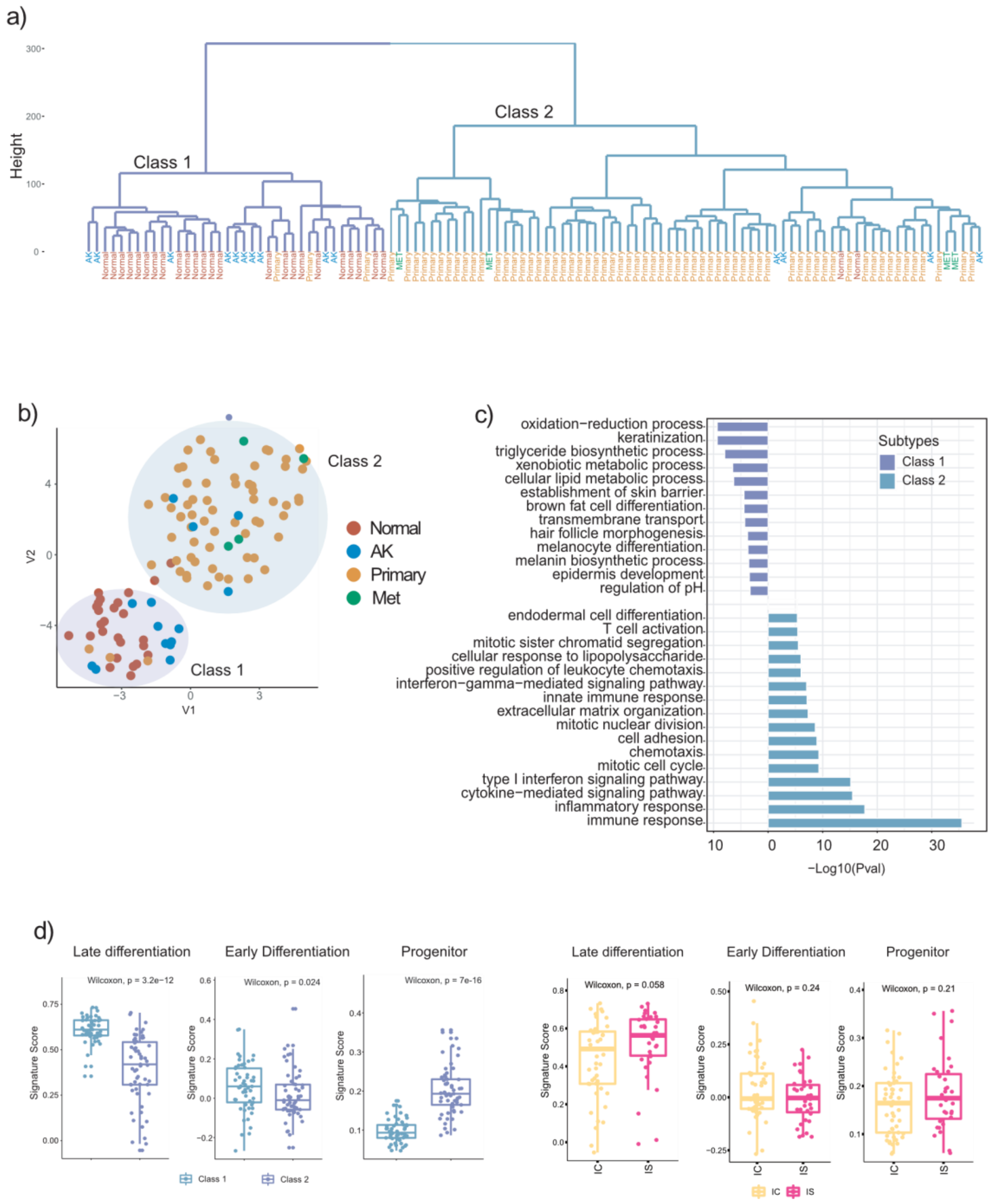

Supplementary Figure 3. Unsupervised clustering of 110 samples from normal skin, AK, primary and metastatic cSCC samples identifies two broad clusters representing Differentiated and Progenitor-like states. **a)** Dendrogram showing hierarchical clustering of normal skin, AK (actinic keratoses), primary and metastatic cSCC samples into two main clusters designated Class1 and Class 2. **b)** tSNE analysis showing two distinct clusters of samples designated as Class 1 and Class 2. Each point represents an individual patient sample and is coloured by clinical designation. **c)** Barplot showing pathways significantly enriched in Class 1 or Class 2. Significance shown as bars -Log10(*P*-values) Fisher's exact test (two-sided) adjusted for multiple testing. **d)** Boxplots showing enrichment of Late Epidermal Differentiation, Early Epidermal Differentiation and Progenitor gene signatures in Class 1 (n=37) and Class 2 (n=73) and in immunocompetent (n=69) and immunosuppressed (n=41) patient samples. Boxplots are annotated by a Kruskall-Wallis P value with P values <= 0.05 indicating a significant difference between sample groups. P-values not adjusted for multiple testing. The boxes visualise the interquartile range (IQR) and median, while whiskers show largest and smallest values within 1.5 * IQR from upper and lower quartiles. Data beyond the whiskers are deemed outliers.

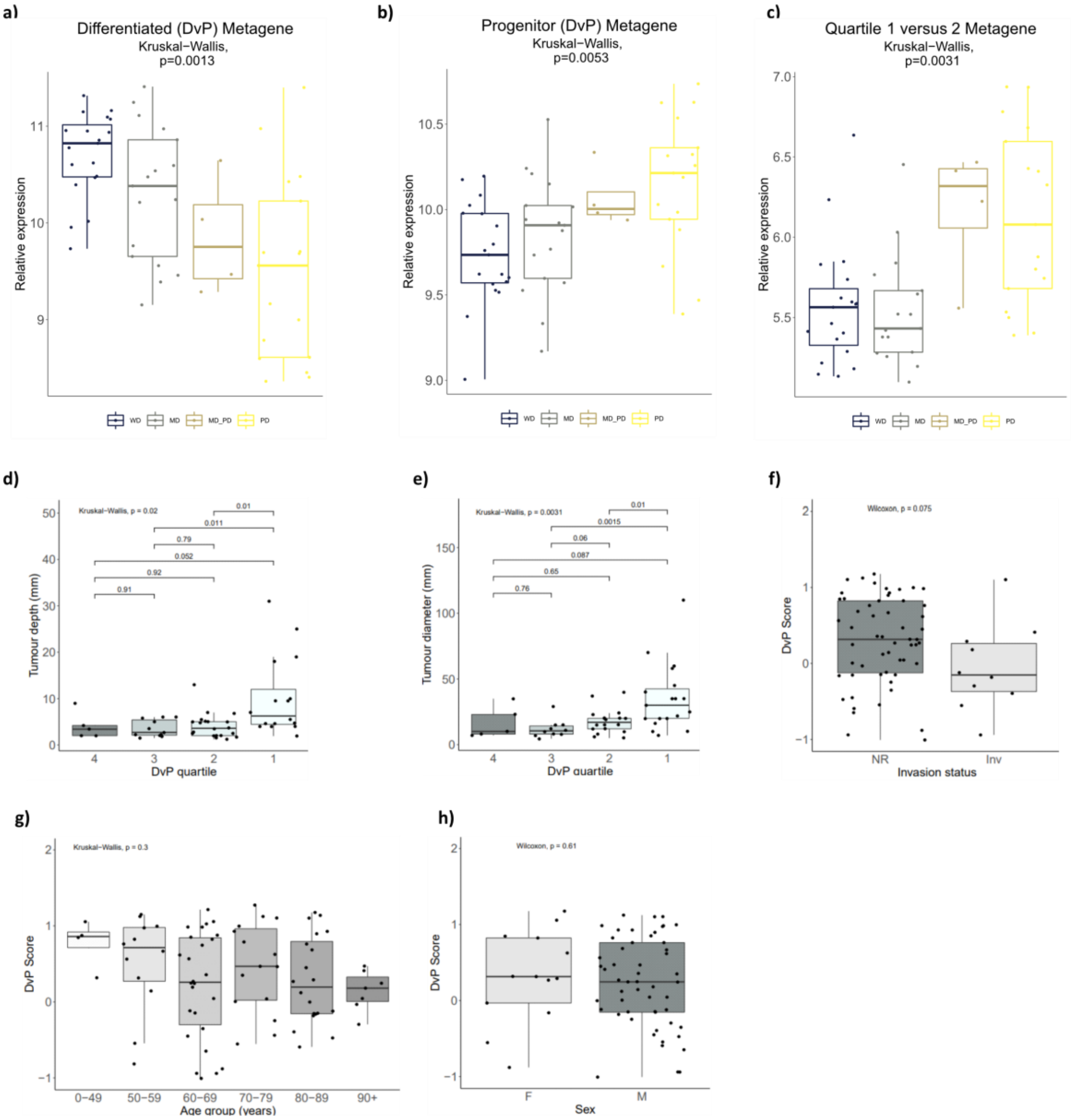

Supplementary Figure 4. Pathological and clinical correlates with DvP metagene, signature score and quartiles. Boxplots showing clinical and pathology parameters in relation to differentiated-progenitor gene expression and quartiles of human primary tumour samples (n=66). Boxplots are annotated by a Kruskall-Wallis P values or Wilcoxon P values as appropriate with P values <= 0.05 indicating a significant difference between sample groups. **a)** Metagene analysis of DP signature genes highly expressed in differentiated samples or **b)** progenitor like samples, correlate with primary tumour differentiation status. **c)** Comparison of genes upregulated (log fold change >1.5, p-value<0.01) in the top quartile (quartile 1) of the DP signature score versus quartile 2 separates MD/PD and PD tumours from MD and WD samples (WD= Well Differentiated n=19, MD=Moderately Differentiated, n=17, PD= Poorly Differentiated, n=17, MD-PD, n=4). **d**) Tumour depth and tumour diameter **e**) increase with progression towards a progenitor like state (number of samples with depth and diameter data is quartile 1 n=16, quartile 2 n=20, quartile 3 n=11, quartile 4 n=5). **f**) DvP signatures score is not significantly different in tumours with annotated perineural or lymphovascular invasion (InV, n=7) when compared to those with these parameters not recorded on the diagnostic pathology records (NR, n=59). DvP signature score does not corelate with age **g)** or sex of patients **h)** (F= female n=13, M= male n=53). Boxplots are annotated by a two-sided Wilcoxon rank-sum test or Kruskal-Wallis test as indicated with P values <= 0.05 indicating a significant difference between sample groups. P-values not adjusted for multiple testing. The boxes visualise the interquartile range (IQR) and median, while whiskers show largest and smallest values within 1.5 * IQR from upper and lower quartiles. Data beyond the whiskers are deemed outliers.

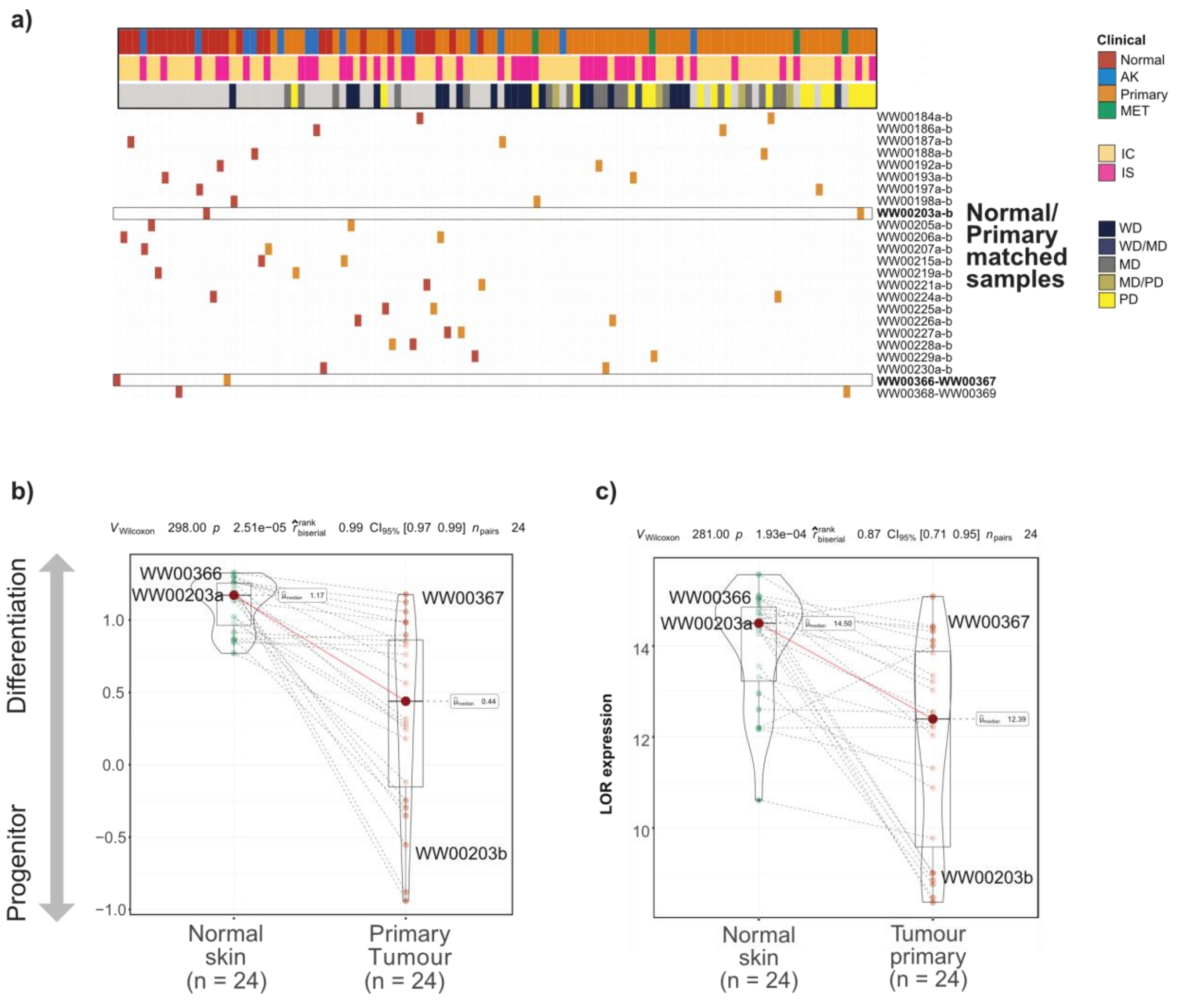

Supplementary Figure 5. Matched analysis of normal and primary pairs. a) Composite figure (comparable to Figure 1b) showing in addition the distribution of normal and primary matched pairs along the Differentiation-Progenitor (DP) axis. Donor IDs are shown on the right hand side of the figure. Representative matched samples (highlighted by a box and in bold) are shown. **b-c)** Paired box plots showing the distributions of matched normal (n=24) and primary (n=24) samples according to D-P score (**b**) or LOR expression (**c**). Dotted lines connect matched samples. The plots are annotated by a Wilcoxon rank sum test with $p < 0.05$ indicating significance. *P*-values were not adjusted for multiple testing. The relative scores of representative matched normal and primary donor pairs (see highlighted samples in panel a) are shown in the plots.

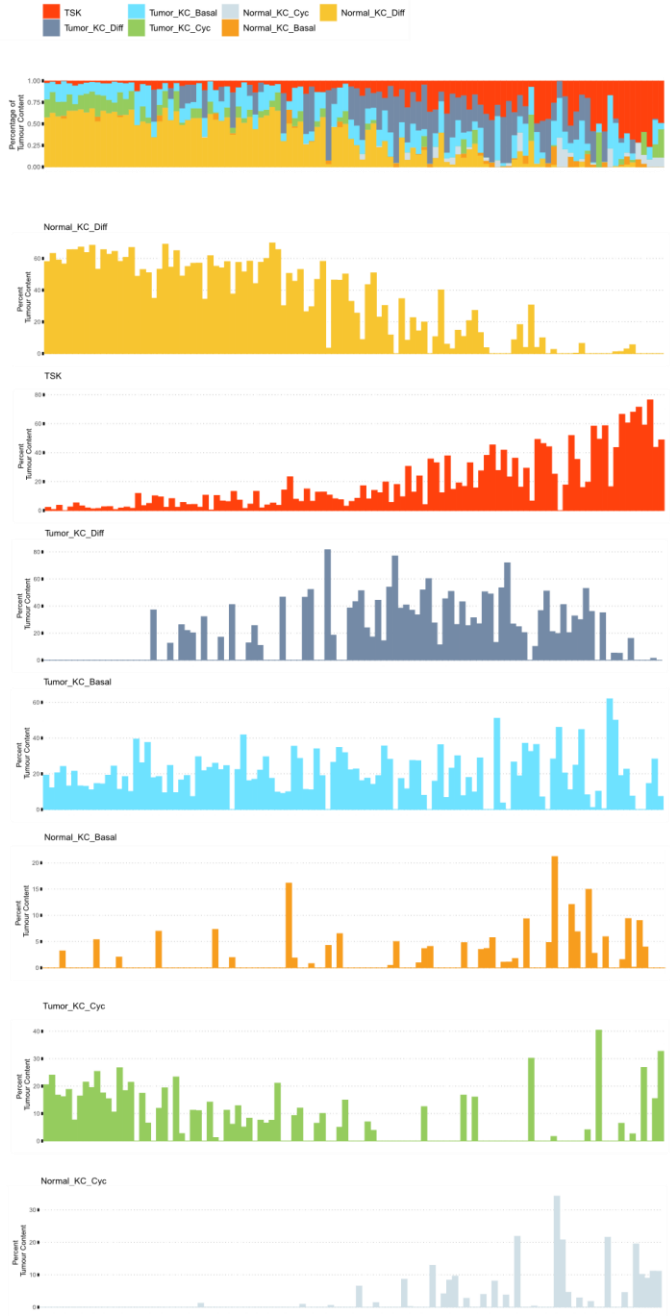

Supplementary Figure 6. CibersortX analysis of keratinocyte populations (Related to Figure 1e). Bar charts showing the enrichment of defined single cell populations in bulk cSCC samples ordered according to the DP signature score (KC, keratinocyte; Diff, differentiated; Cyc, cycling).

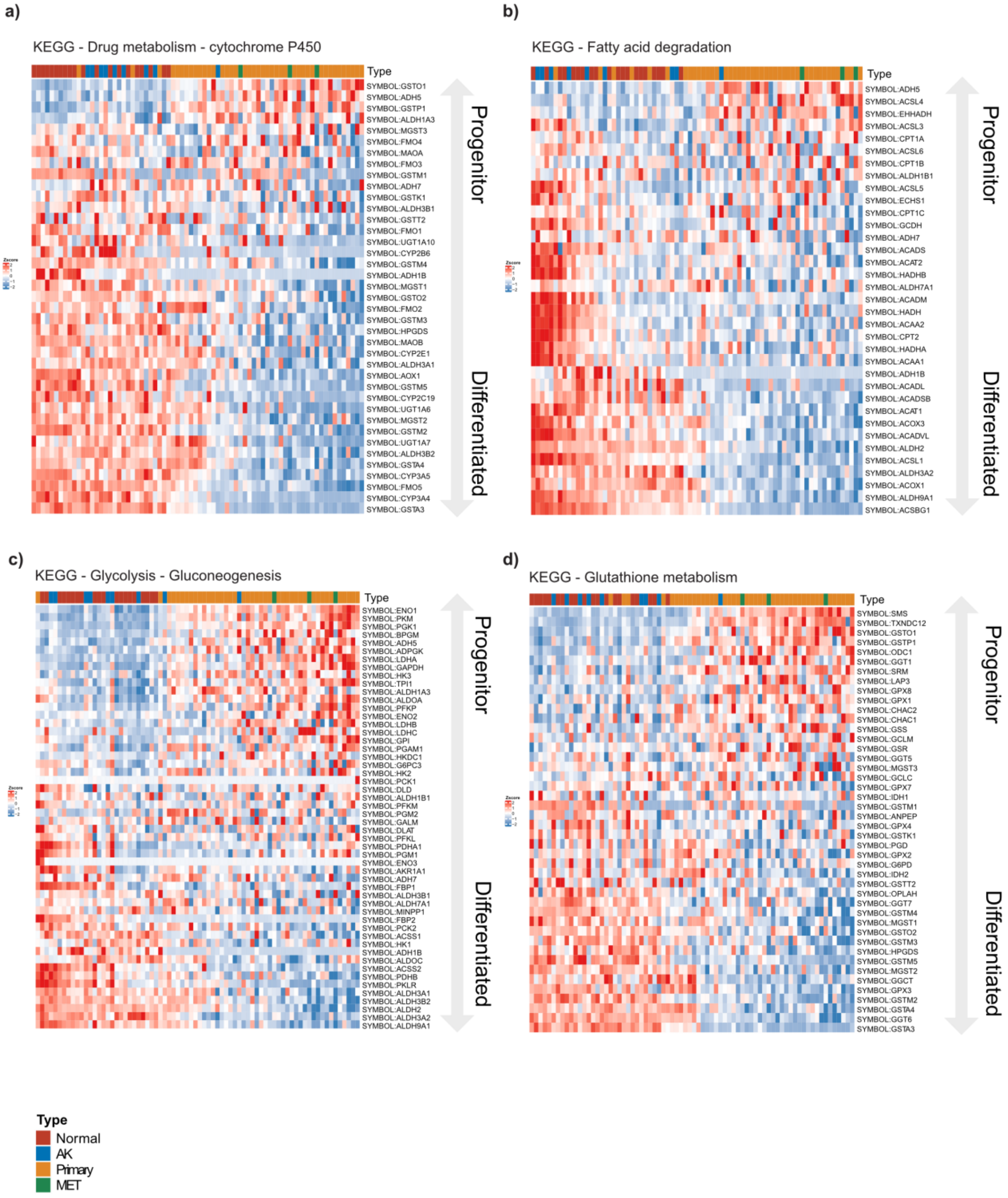

Supplementary Figure 7. Metabolic gene alterations during disease progression. a-d) Heatmaps of selected KEGG metabolic pathways showing correlated changes in gene expression between Normal, AK, Primary and MET samples. Genes are ranked according to significant high progenitor-like expression (top of heatmap) versus high differentiated expression (bottom of heatmap).

a)

b)

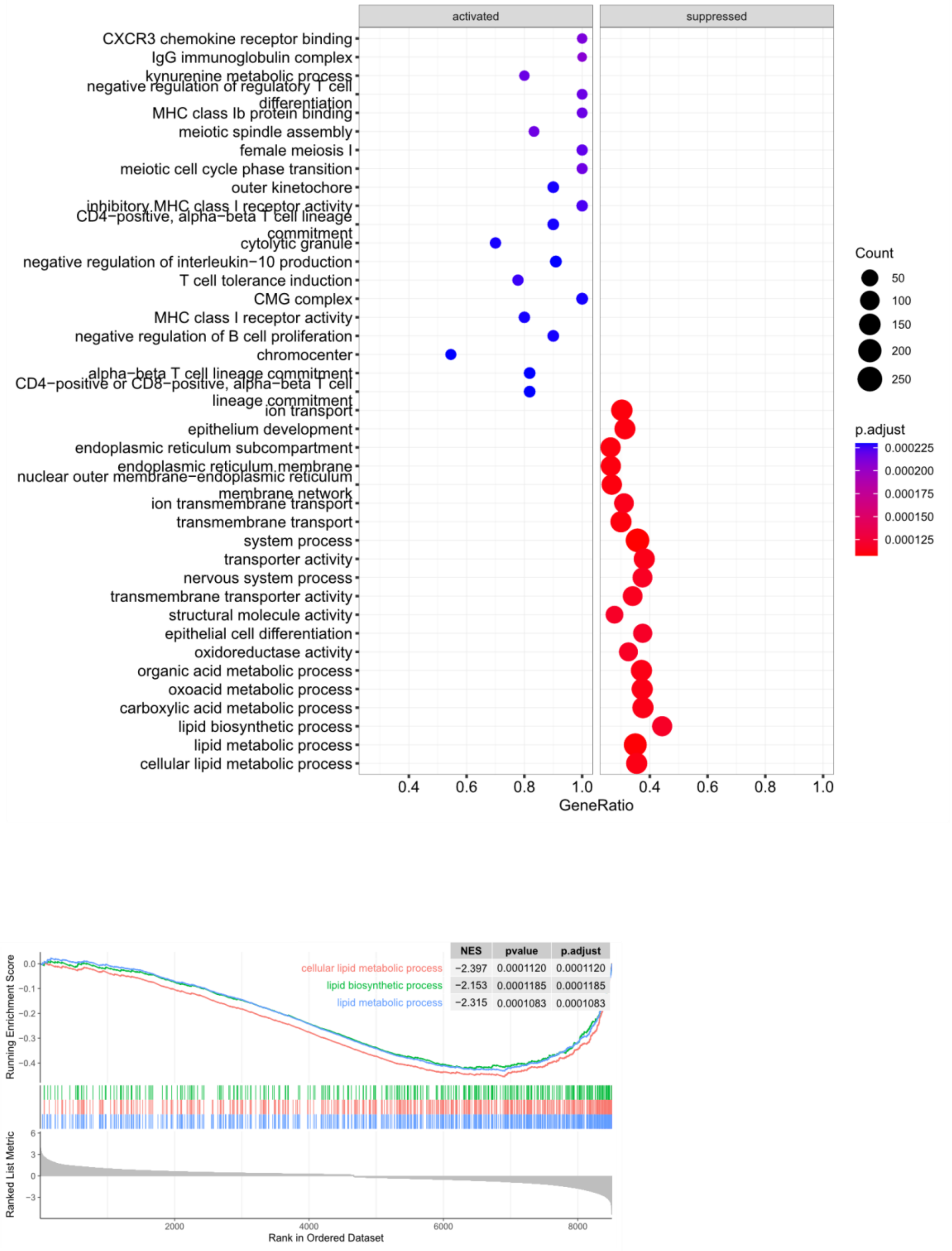

Supplementary Figure 8. Gene ontology and gene set enrichment analysis comparing progenitor like samples to differentiated samples. a) Gene ontology analysis of top 20 activated (expressed more highly in progenitor like samples) and top 20 suppressed (expressed more highly in differentiated like samples) generated from differential gene expression analysis (p-adjust <0.01) comparing quartiles 1 and 2 to quartiles 3 and 4. **b)** GSEA analysis of lipid metabolic and biosynthetic processes. Plots annotated by one-sided p-values from Fisher's test (labelled as p.adjust) by the clusterProfiler R package. P values <= 0.05 indicating a significant difference between sample groups. P-values not adjusted for multiple testing.

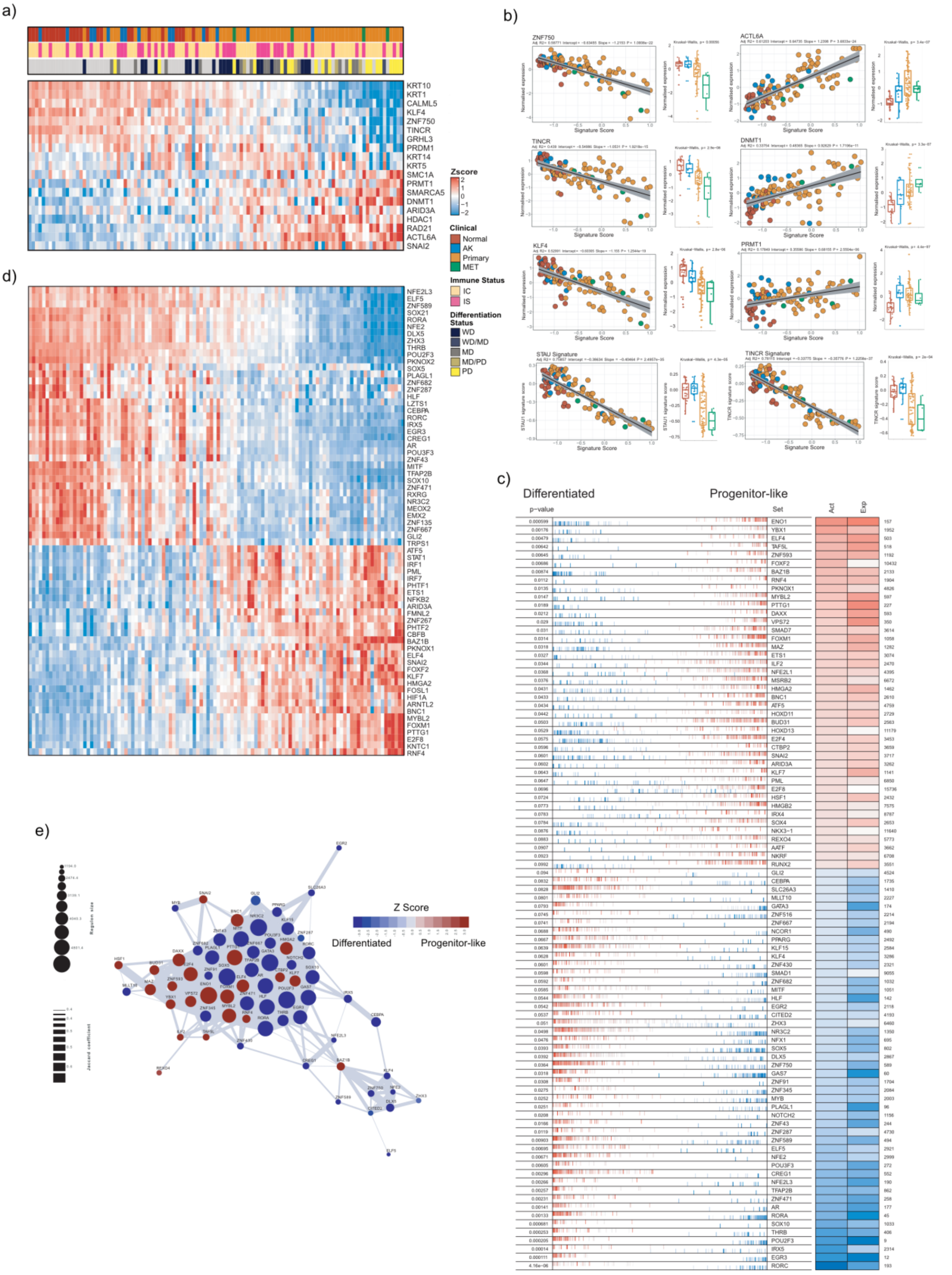

Supplementary Figure 9. The orchestrated suppression of late epidermal differentiation and induction of progenitor-like gene expression is mediated by master regulators of epidermal differentiation. a) Heatmap

showing the relative gene expression of previously characterised master regulators of epidermal differentiation. Samples are ordered by DP gene signature score. **b)** Scatter plots showing correlations between master regulator gene expression values and the DP signature score. Each point represents an individual and is coloured by clinical designation (normal n=26, AK n=14, primary n=66, Met n=4). Box plots show the expression of each master regulator stratified by clinical designation. Box plots are annotated by a Kruskall-Wallis P value with P values <= 0.05 indicating a significant difference between clinical designations. The boxes visualise the interquartile range (IQR) and median, while whiskers show largest and smallest values within 1.5 * IQR from upper and lower quartiles. Data beyond the whiskers are deemed outliers. **c)** msViper plot showing the differential enrichment of TF regulons between the Differentiated and Progenitor-like states. The two colour heatmap displayed on the right of the plot represents the inferred differential activity of each TF regulon (first column) and differential expression (second column) - red denotes TF regulons enriched in the Progenitor-like state while blue denotes TF regulons down-regulated in the Progenitor-like state. **d)** Heatmap showing the differential enrichment of Transcription Factor (TF) regulon activity scores between Differentiated and Progenitor-like states. **e)** Network showing TF regulons enriched in cSCC. TF regulons enriched in Differentiated cSCC are shown as blue circles whereas TF regulons enriched in Progenitor-like cSCC are shown in red. The size of each TF regulon is represented by the size of the circle and the overlap between TF regulons is measured by the Jaccard coefficient (JC) which is represented by edge size. Only regulons with JC >= 0.4 are shown.

a)

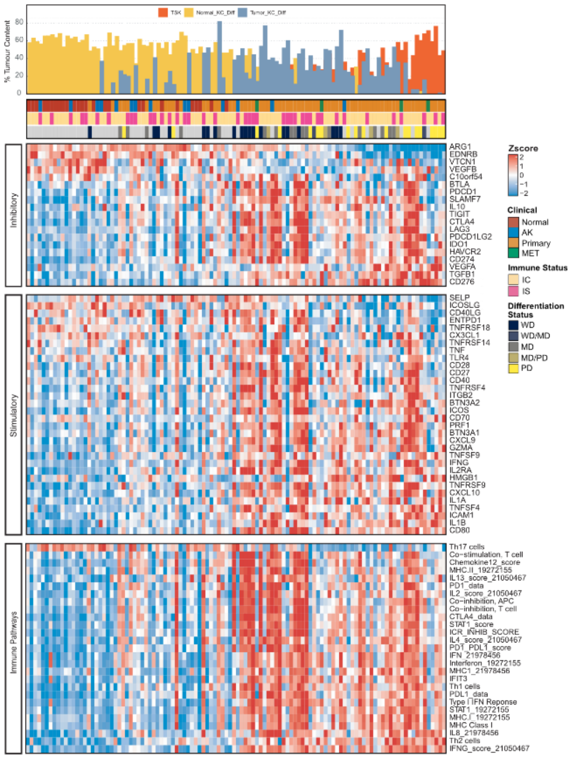

b)

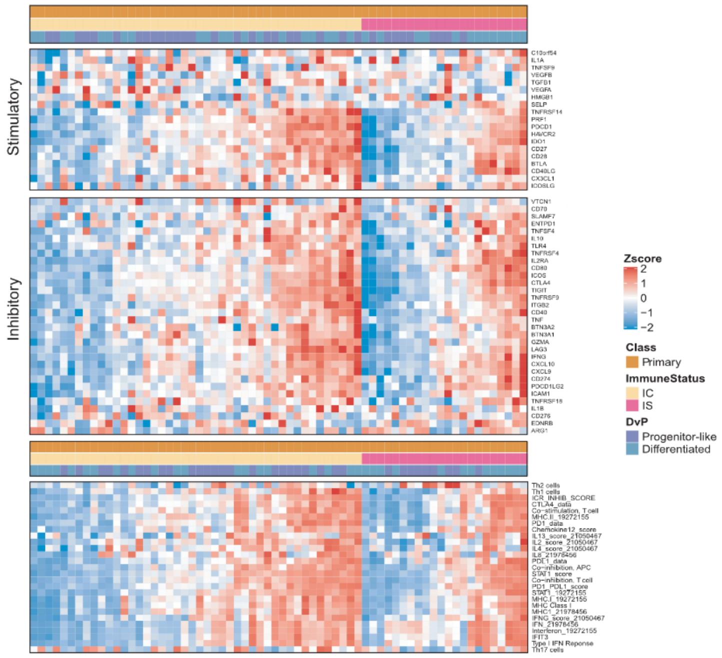

Supplementary Figure 10. Modulation of the immune landscape in cSCC disease progression. **a)** Heatmap showing the expression of immunomodulatory genes and the enrichment scores of selected immune pathways in bulk RNAseq samples. Patient samples are ordered by DP signature score and the relative tumour enrichment of single cell defined tumour cell types is shown in the top bar chart. Samples are ordered by DP signature score. **b)** Bulk RNaseq analysis of primary tumour samples and immunomodulatory genes (Stimulatory, and inhibitory) and selected immune pathways (bottom) that are significantly enriched in immunocompetent (IC) versus immunosuppressed (IS) patient groups. Patient samples are ordered by sample group and column sum (i.e. sum of all values in a given patient sample) in all heatmaps.

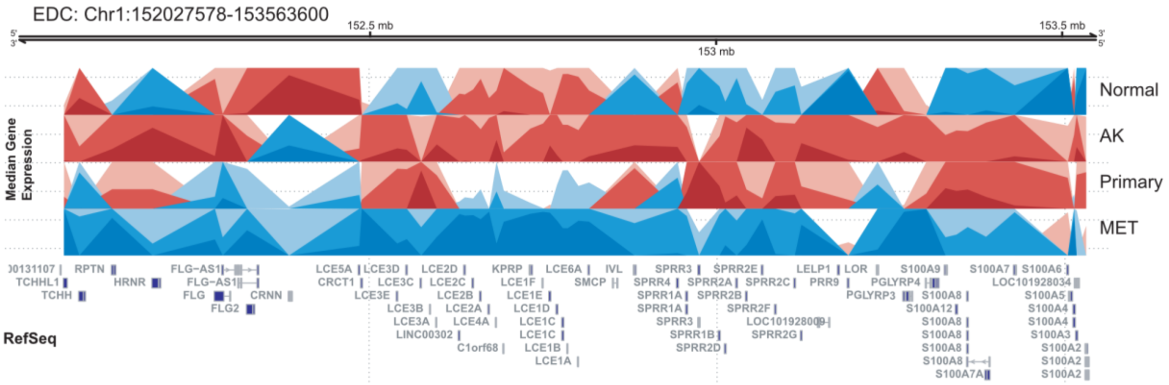

Supplementary Figure 11. Modulation of the Epidermal Differentiation Complex in cSCC disease states. Horizon plot showing the relative expression of EDC genes at different tumour stages. Red represents increased gene expression whereas blue represents downregulation of gene expression.

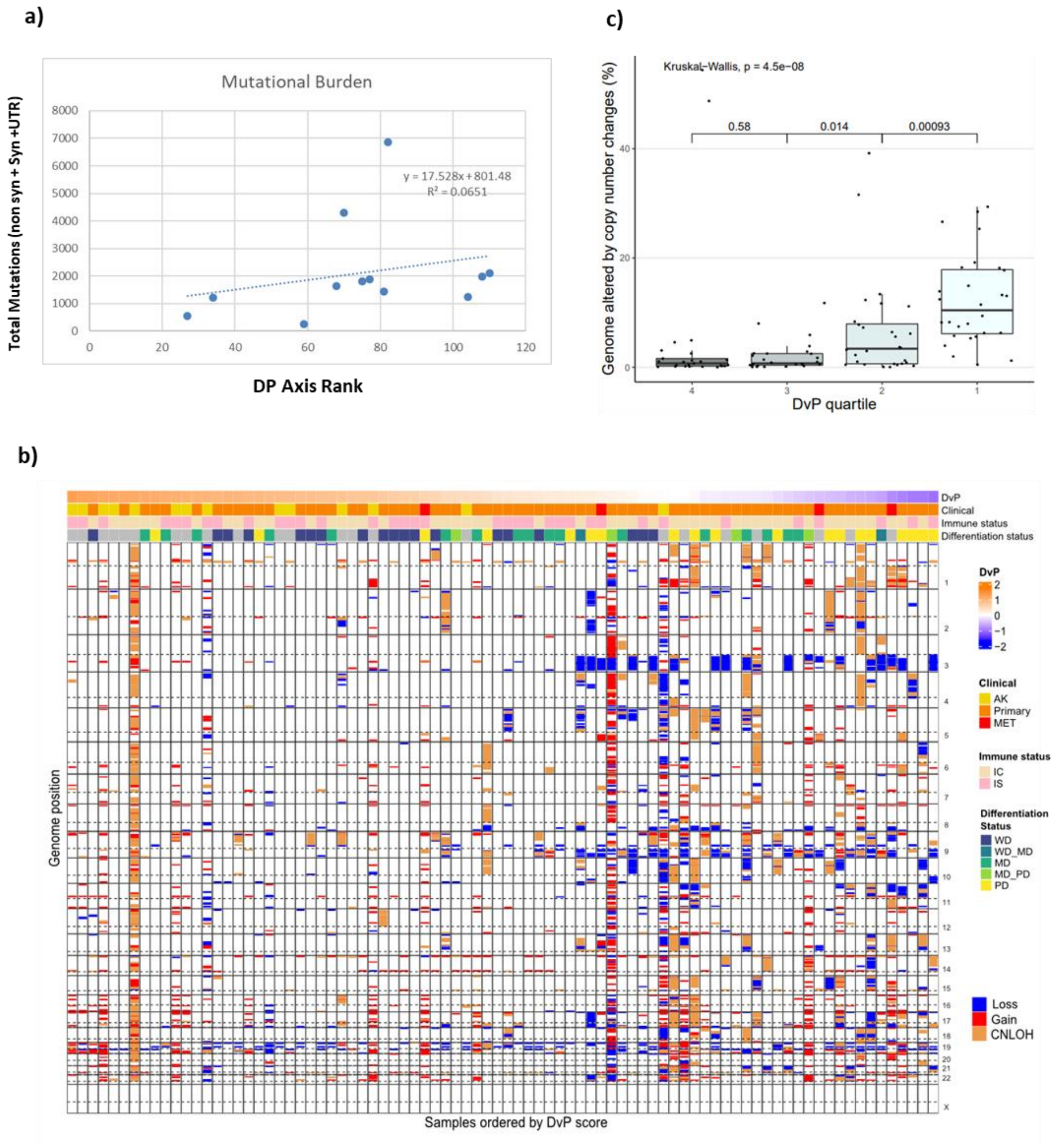

Supplementary Figure 12. Correlation of mutational load and copy number alterations in cSCC with DP axis rank. a) Scatter plot of total mutational burden (non-synonymous + synonymous and UTR mutations) of samples previously profiled by WES[11] versus DP axis rank. **b)** Copy number variations estimated from bulk RNAseq data utilising CaSpER[33]. Samples (AK n=24, primary SCC n=66, Metastasis n=4)) are ordered by DP axis and regions of copy number loss, gain and copy number neutral loss of heterozygosity are indicated. **c)** Box plot of the % of genome altered by CNV by DP quartile. The boxes visualise the interquartile range (IQR) and median, while whiskers show largest and smallest values within 1.5 * IQR from upper and lower quartiles. Data beyond the whiskers are deemed outliers.

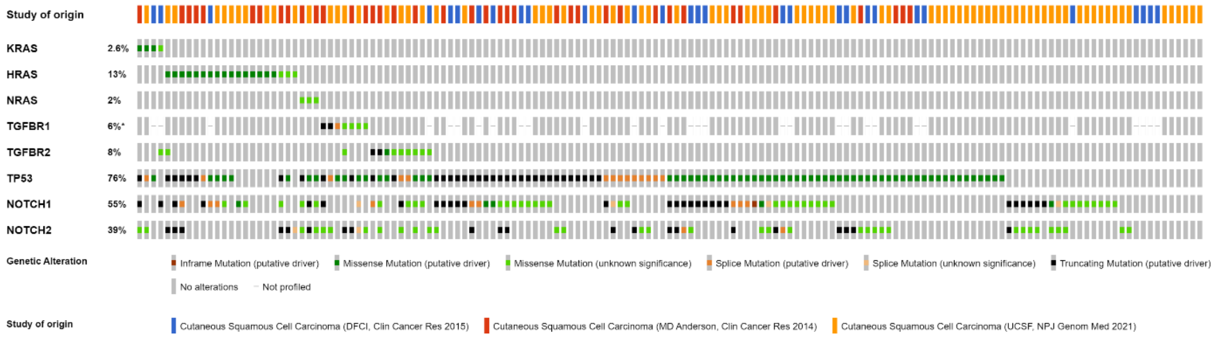

Supplementary Figure 13. Oncoprint analysis of cSCC driver genes. Cbioportal analysis[34-35] of indicated genes from 151 cSCC samples collated from the indicated studies.

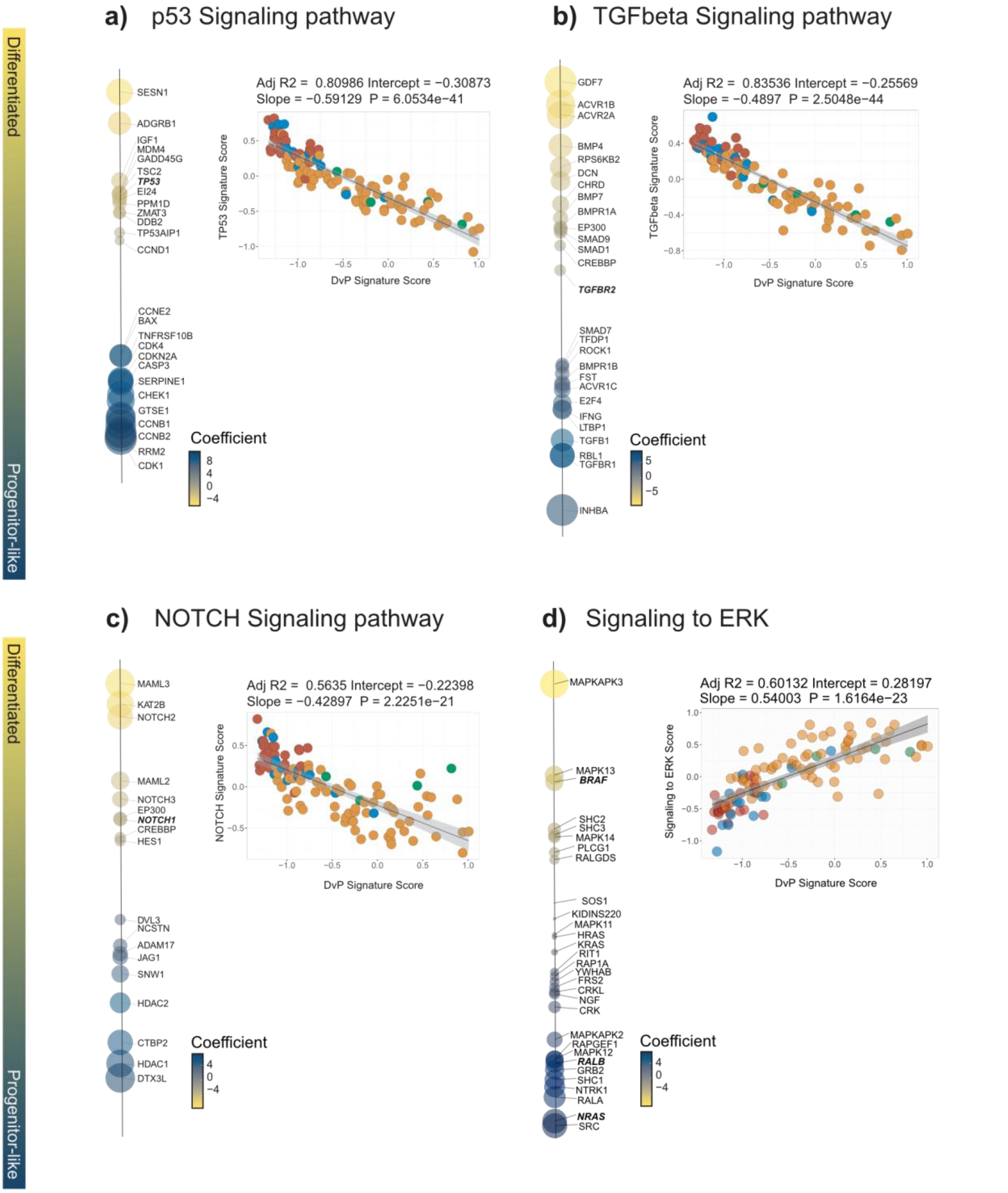

Supplementary Figure 14. Dysregulation of p53, TGFβ, NOTCH and ERK signalling pathways during cSCC disease progression. a-d) Bubble plots showing the differential expression of genes comprising the p53, TGFβ, NOTCH, and ERK signalling pathways. The genes indicated show significant differential expression between the Differentiated and Progenitor-like state and are ordered by Log fold change. The size of each circle (i.e., gene) is indicative of the log fold change. Scatter plots show the correlation between signalling pathway sample enrichment and DP signature score. Each point represents an individual patient sample and is coloured by cSCC clinical designation (red=NSE, blue=AK, Orange=primary tumour, green=metastasis). Pearson's correlations are shown in the plots. Significance was determined by two-sided Pearson's correlation test. *P*-values were not adjusted for multiple testing. The plots show a solid regression line and error bands representing 95% confidence intervals.

a) LPT

b) LNPT

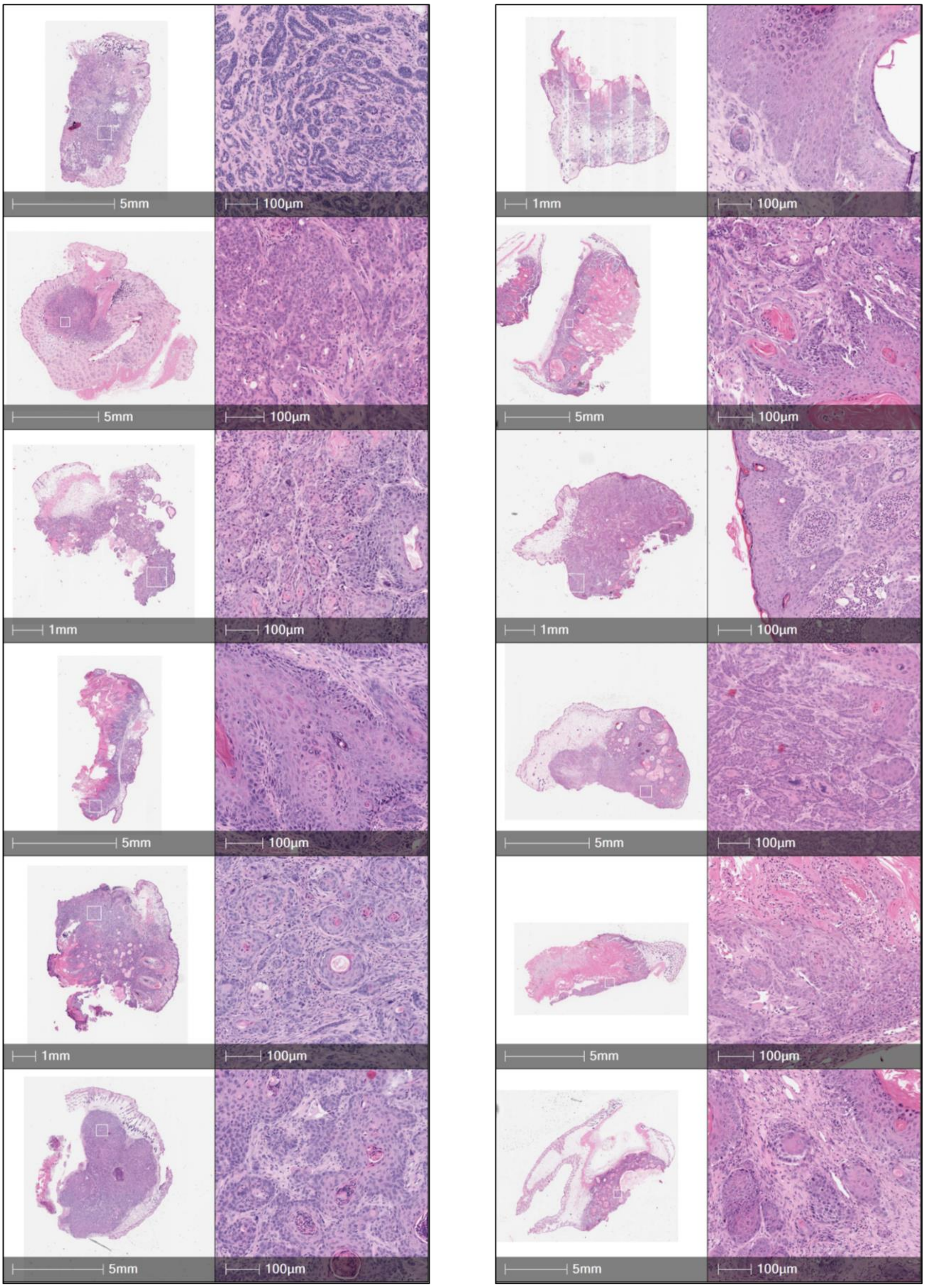
5mm
100µm
5mm
100µm
1mm
100µm
5mm
100µm
1mm
100µm
5mm
100µm
1mm
100µm
5mm
100µm
1mm
100µm
5mm
100µm
5mm
100µm
5mm
100µm

c) **LKT**

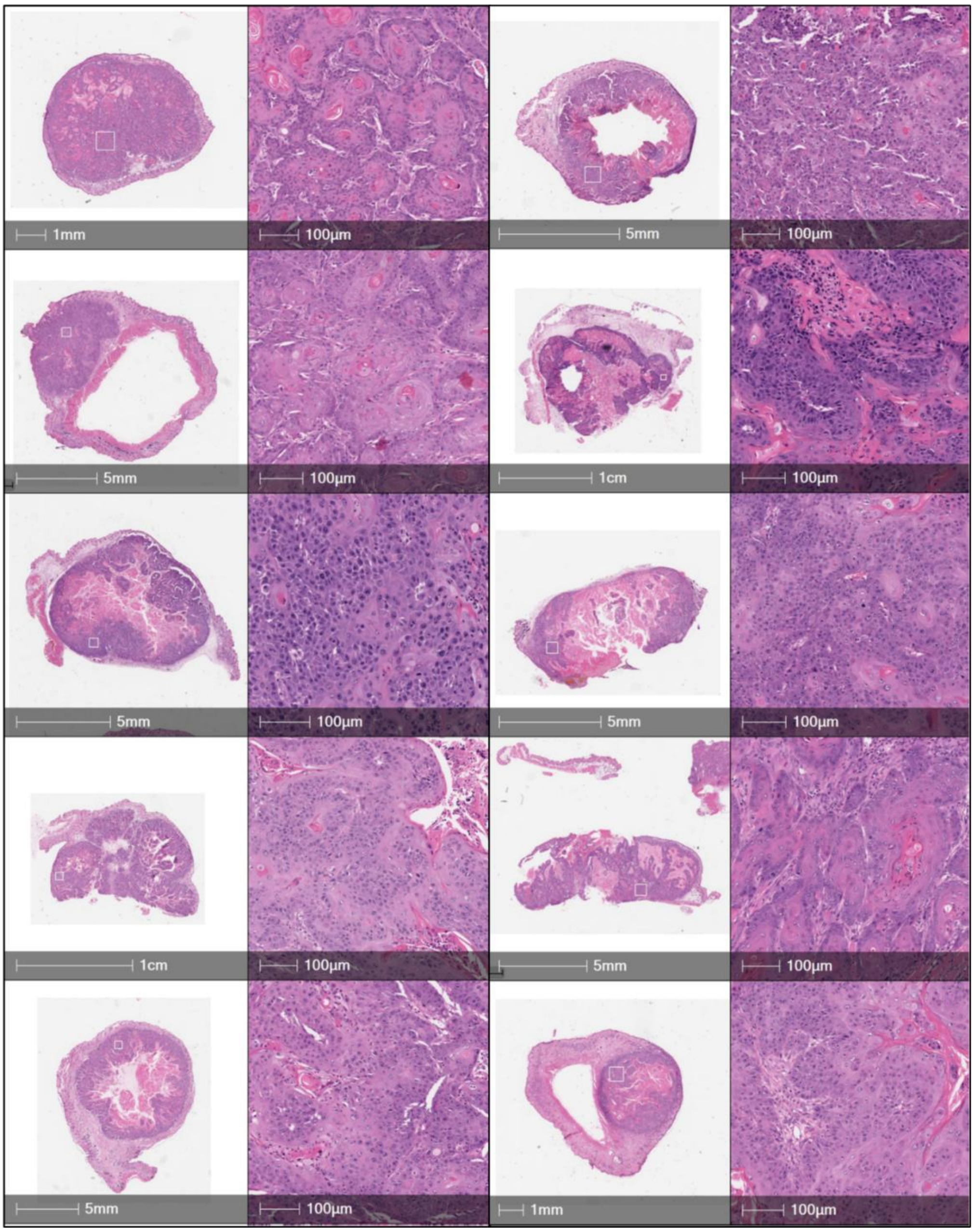

Supplementary Figure 15. H&E staining of murine tumours. Images of H&E staining of selected murine tumours isolated from the indicated genotypes (L=*Lgr5*, P=*Trp53*, T=*Tgfbr2,* N=*Notch1,* K= Kras^{G12D}):- **a)** LPT, **b)** LNPT, **c)** LKT. Full images of tumours are shown in the left hand columns, next to zoomed in images of the selected white boxed regions in the right hand columns. Scale bars are shown in each image.

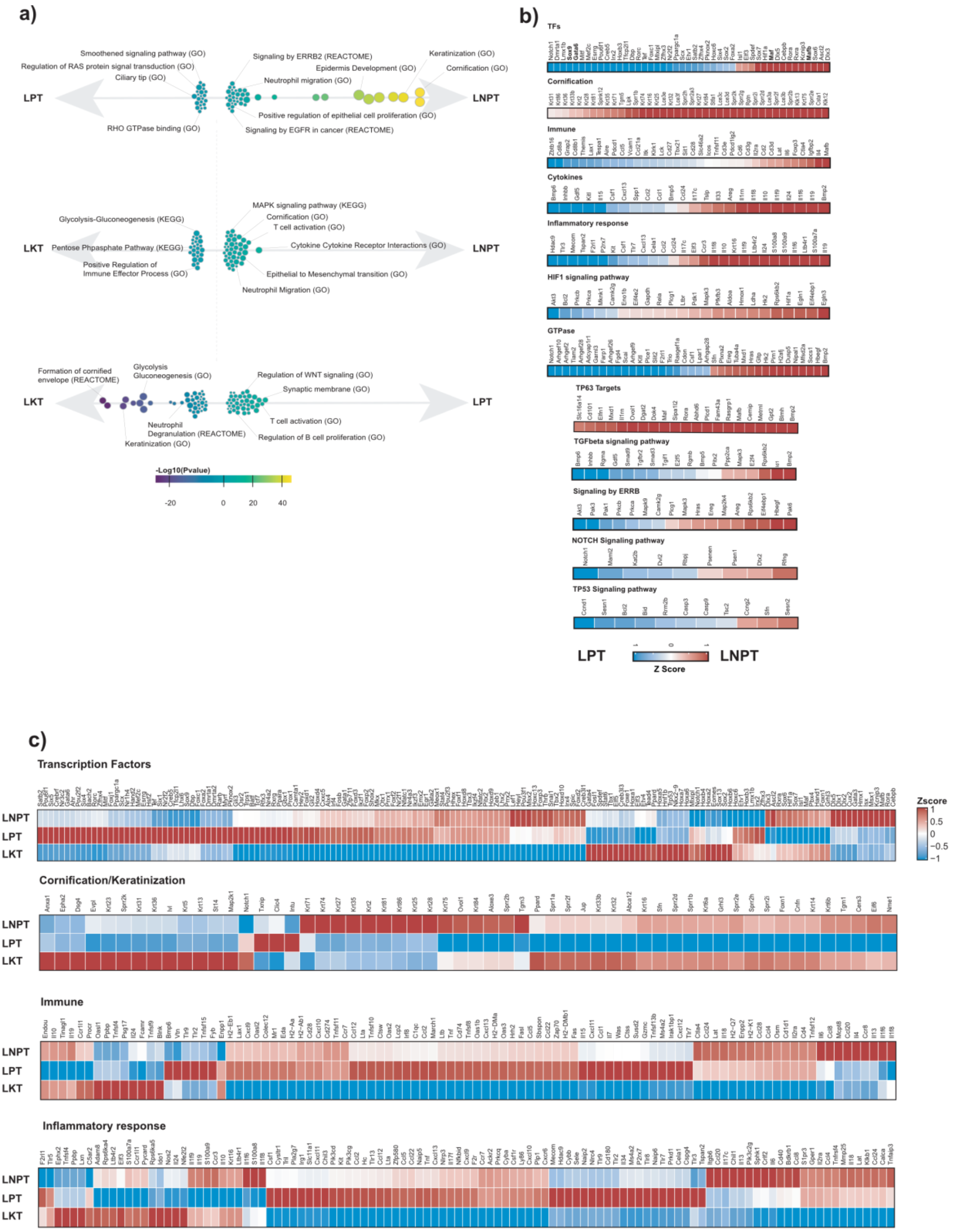

a)
LPT
LNPT
Smoothened signaling pathway (GO)
Regulation of RAS protein signal transduction (GO)
Ciliary tip (GO)
RHO GTPase binding (GO)
Signaling by ERRB2 (REACTOME)
Epidermis Development (GO)
Neutrophil migration (GO)
Keratinization (GO)
Cornification (GO)
Positive regulation of epithelial cell proliferation (GO)
Signaling by EGFR in cancer (REACTOME)
LKT
Glycolysis-Gluconeogenesis (KEGG)
Pentose Phpasphate Pathway (KEGG)
Positive Regulation of Immune Effector Process (GO)
MAPK signaling pathway (KEGG)
Cornification (GO)
T cell activation (GO)
Cytokine Cytokine Receptor Interactions (GO)
Epithelial to Mesenchymal transition (GO)
Neutrophil Migration (GO)
Formation of cornified envelope (REACTOME)
Glycolysis Gluconeogenesis (GO)
Neutrophil Degranulation (REACTOME)
Keratinization (GO)
Regulation of WNT signaling (GO)
Synaptic membrane (GO)
T cell activation (GO)
Regulation of B cell proliferation (GO)
-Log10(Pvalue)
-20
0
20
40
b)
TFs
Cornification
Immune
Cytokines
Inflammatory response
HIF1 signaling pathway
GTPase
TP63 Targets
TGFbeta signaling pathway
Signaling by ERRB
NOTCH Signaling pathway
TP53 Signaling pathway
LPT
LNPT
Z Score
c)
Transcription Factors
LNPT
LPT
LKT
Zscore
1
0.5
0
-0.5
-1
Cornification/Keratinization
Immune
Inflammatory response

d)

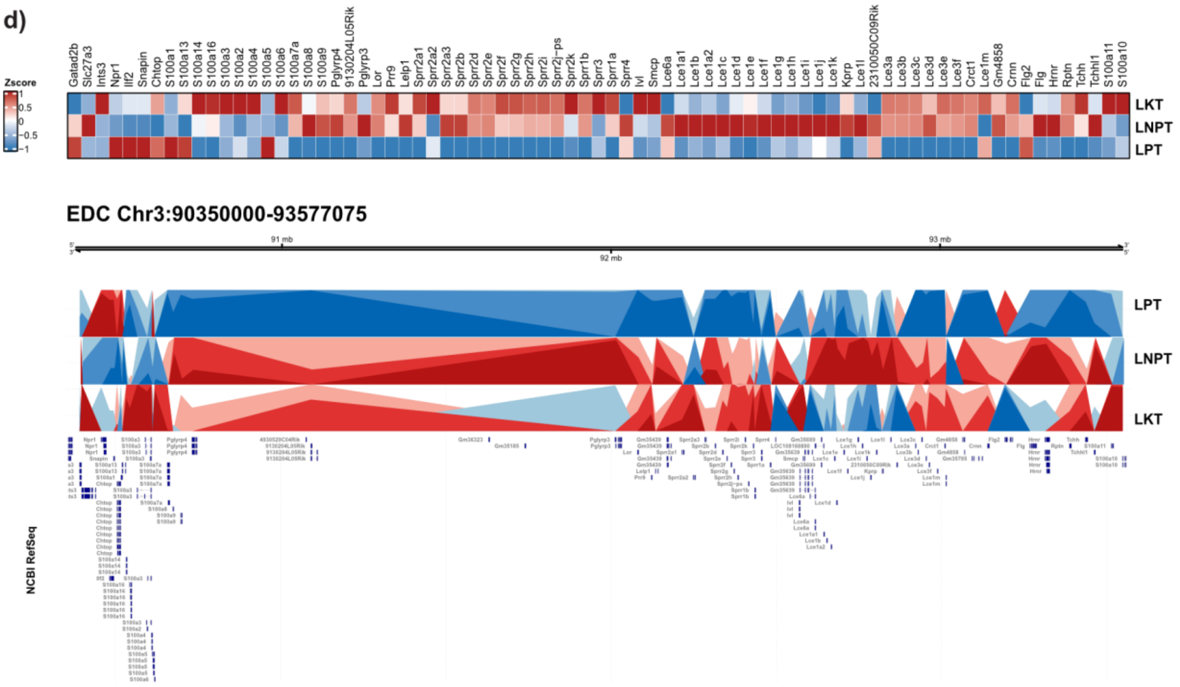

Supplementary Figure 16. Modulation of biological processes, differentiation and the immune landscape in GEMM cSCC models. a) Swarm plot showing pathways significantly enriched in GEMMs (L=*Lgr5*, P=*Trp53*, T=*Tgfbr2,* N=*Notch1,* K= Kras^{G12D}). For each comparison the x-axis represents -log10(P value) of enrichment scores with node size and colour providing additional indications of pathway significance. Pathways of significant biological and clinical importance are labelled. **b)** Heatmaps showing mean expression of genes that are significantly and differentially expressed between LPT versus LNPT GEMMs. Sets of genes are categorised by biological function and/or process. **c)** Heatmaps showing mean expression of transcription factors, cornification/keratinization, immune and inflammatory response genes that are significantly and differentially expressed between GEMMs. **d)** Heatmaps showing mean expression of murine EDC genes that are significantly and differentially expressed between the indicated GEMMs (upper panel) and Horizon plot (lower panel) showing the relative expression of EDC genes in the indicated GEMMs. Red represents increased gene expression whereas blue represents downregulation of gene expression.

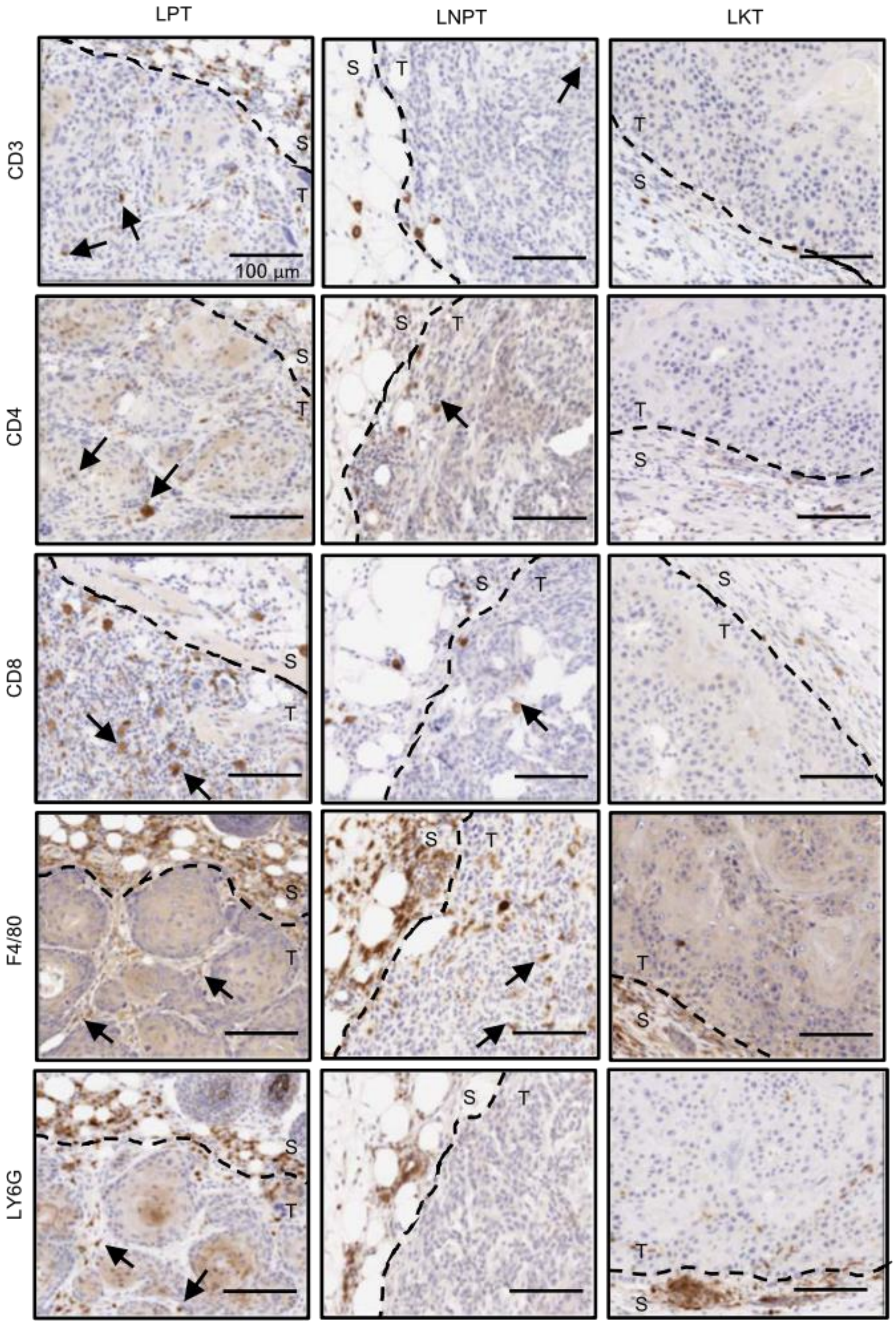

Supplementary Figure 17. Immunohistochemical analysis of GEMM tumours. Representative images of IHC for the indicated markers in tumours isolated from the indicated murine tumour genotypes (L=*Lgr5*, P=*Trp53*, T=*Tgfbr2,* N=*Notch1,* K= $Kras^{G12D}$), (LPT, n=6; LNPT, n=6, LKT, n=10). Arrows highlight positive cells and dotted lines indicate borders between islands of tumour cells (T) and surrounding stroma (S). Scale bar=100μm.

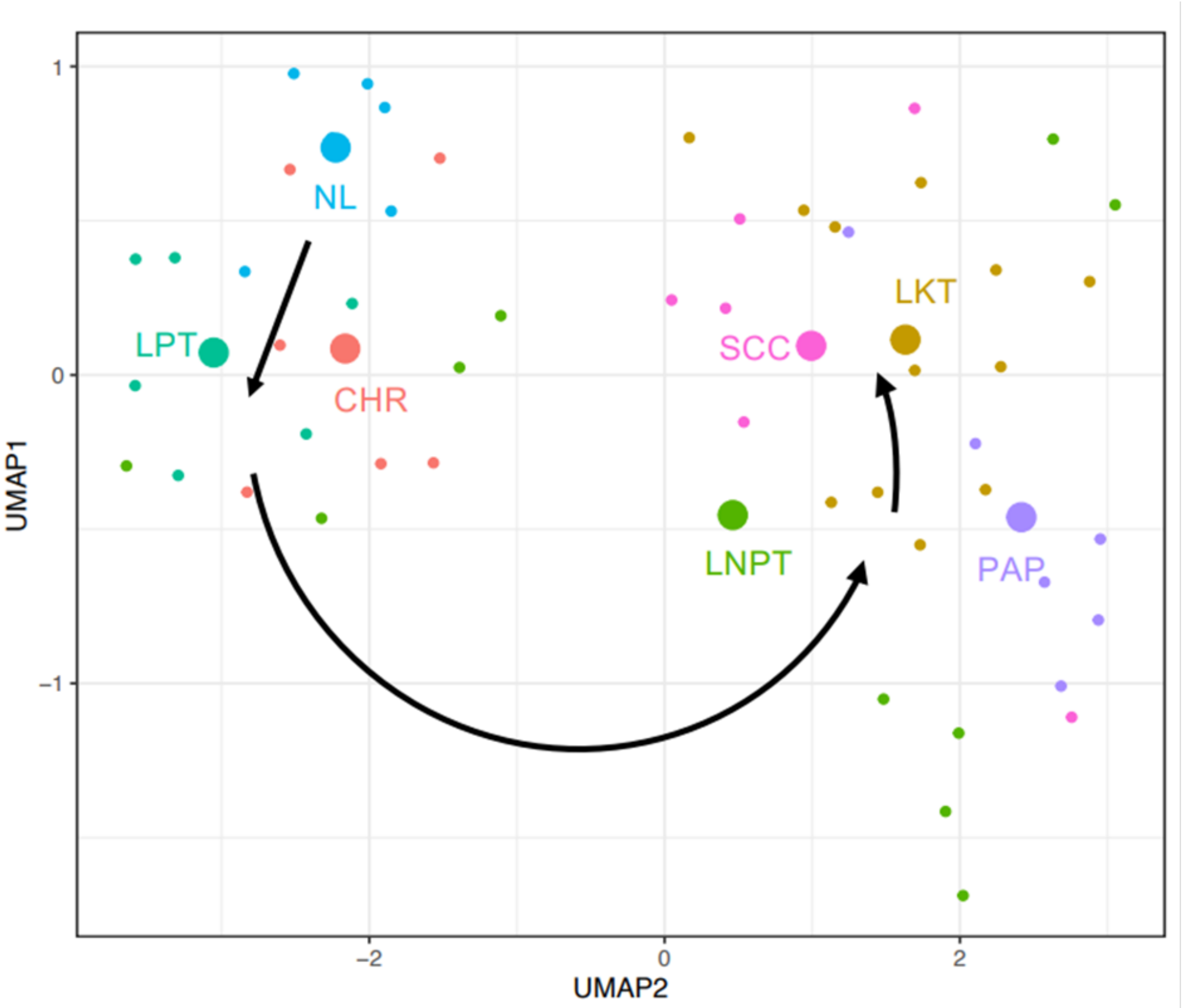

Supplementary Figure 18. UMAP of GEMM and UV induced mouse model transcriptomic data showing proximal clustering of samples. A Uniform Manifold Approximation and Projection (UMAP) reveals the proximity of sample transcriptomic data clusters from the 3 generated GEMM mouse models (LPT, LNPT, LKT; L=*Lgr5*, P=*Trp53*, T=*Tgfbr2,* N=*Notch1,* K= Kras^{G12D}) and 4 UV induced mouse sample types[8]; normal (NL)(n=6), chronically irradiated skin (CHR)(n=6), precancerous papillomas (PAP)(n=6), and cutaneous cSCC (SCC)(n=6). Smaller coloured points correspond to individual sample data from each matched designation colour. Mean points of each sample designation are presented as larger coloured circles joined by black arrows which indicate the direction of disease progression.

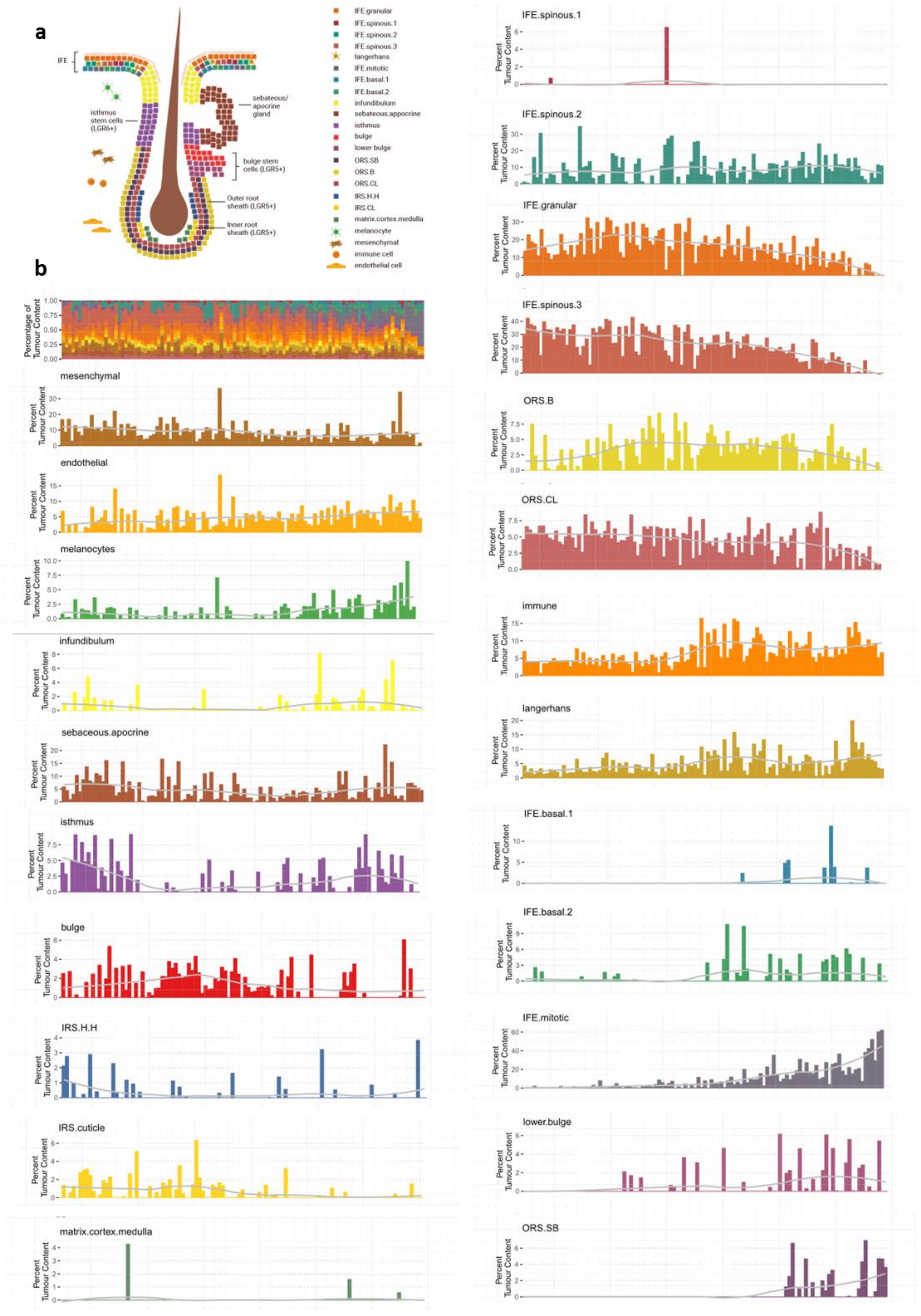
a
IFE
isthmus stem cells (LGR6+)
sebateous/ apocrine gland
bulge stem cells (LGR5+)
Outer root sheath (LGR5+)
Inner root sheath (LGR5+)
IFE.granular
IFE.spinous.1
IFE.spinous.2
IFE.spinous.3
langerhans
IFE.mitotic
IFE.basal.1
IFE.basal.2
infundibulum
sebateous.appocrine
isthmus
bulge
lower bulge
ORS.SB
ORS.B
ORS.CL
IRS.H.H
IRS.CL
matrix.cortex.medulla
melanocyte
mesenchymal
immune cell
endothelial cell
b
Percentage of Tumour Content
mesenchymal
endothelial
melanocytes
infundibulum
sebaceous.apocrine
isthmus
bulge
IRS.H.H
IRS.cuticle
matrix.cortex.medulla
IFE.spinous.1
IFE.spinous.2
IFE.granular
IFE.spinous.3
ORS.B
ORS.CL
immune
langerhans
IFE.basal.1
IFE.basal.2
IFE.mitotic
lower.bulge
ORS.SB
Percent Tumour Content

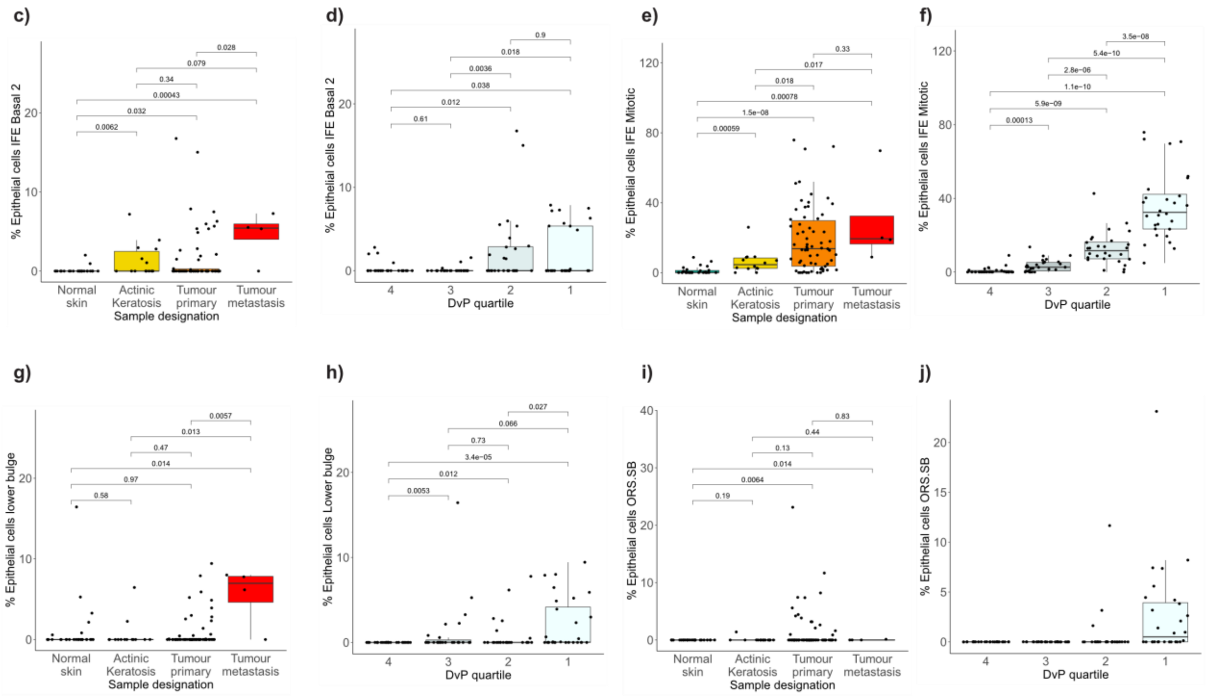

Supplementary Figure 19. SSGSEA analysis of normal human skin and hair follicle single cell signatures. a) Diagram of 23 human skin and hair follicle cell states adapted from[43]. IFE, (interfollicular epidermis), ORS (outer root sheath), IRS (inner root sheath). Deconvolution of bulk cSCC RNAseq data using human hair follicle cell state transcriptional signatures[43] was performed using the Gene expression deconvolution interactive online tool (GEDIT) found at: http://webtools.mcdb.ucla.edu (Default settings). **b)** Cell state enrichment scores for each patient sample were plotted in a barplot either together (relative enrichment) or individually. **c-j)** Boxplots of % of epithelial cells containing IFE Basal2 **(c,d),** IFE Mitotic **(e,f)**, lower bulge **(g,h)** and ORS-SB **(I,j)** signatures across disease states **(c,e,g,i**; normal n=26, AK n=14, primary n=66, Met n=4**)** and DvP quartiles **(d,f,h,j**; q1 n=27, q2 n=27, q3 n=28, q4 n=28**)**. Boxplots are annotated by two-sided Wilcoxon rank-sum tests with P values <= 0.05 indicating a significant difference between sample groups. P-values not adjusted for multiple testing. The boxes visualise the interquartile range (IQR) and median, while whiskers show largest and smallest values within 1.5 * IQR from upper and lower quartiles. Data beyond the whiskers are deemed outliers.
