# Supplementary material for: Driver gene combinations dictate cutaneous squamous cell carcinoma disease continuum progression
Source: Nat Commun. 2023 Aug 25;14:5211. doi: 10.1038/s41467-023-40822-9 (PMC10457401; doi:10.1038/s41467-023-40822-9)
Supplement: Supplementary file 3 — Description of Additional Supplementary Files [file 41467_2023_40822_MOESM3_ESM.docx]

**Supplementary Inventory.**

**Supplementary Figures** 1-19 supplied as Supplementary Figure File

**Supplementary Data**

**Supplementary Data File 1.** Data supporting Figure 1, including: Clinical manifest including patient and tumour characteristics from the clinical records and pathology reports; RNAseq merged count data and LogR normalised gene expression values.

**Supplementary Data File 2.** Data supporting Supplementary Figure 1, including differential gene expression analyses and GSEA analyses. Differential gene expression tables of the following comparisons between clinical designations: Normal versus Rest; Normal versus Primary, Normal versus AK, AK versus Rest, Primary versus AK, and Primary versus Rest. GSEA tables of the following comparisons, Normal versus AK up, Normal versus AK down, Normal versus Primary down, Normal versus Primary up, Primary versus AK up and Primary versus AK down.

**Supplementary Data File 3.** Data supporting Supplementary Figure 2 including differential gene expression analysis of matched primary versus normal samples and GSEA reactome terms of this comparison.

**Supplementary Data File 4.** Data supporting Supplementary Figure 3a-c including classification of samples based on top 2000 most variable genes; Differentially expressed genes between Class 1 and Class 2; Significantly enriched ontologies for Class 1; Significantly enriched ontologies for Class 2.

**Supplementary Data File 5.** Data supporting Figure 1a-c and Supplementary Figure 3d including curated Early differentiation signature genes, Late differentiation signature genes, Progenitor signature genes[16], Early-late-progenitor signature scores for each sample, the DvP signature genes and their coefficients and the DvP signature score for each sample.

**Supplementary Data File 6.** Data supporting Figure 1d-g and Supplementary Figure 6 including CIBERSORTx scores and CIBERSORTx scores ordered by DVP score.

**Supplementary Data File 7.** Data Supporting Figure 2a-b including tables of genes for the 15 clusters of co-ordinately expressed genes identified by k-means clustering, GSEA using curated Reactome pathways showing significantly enriched molecular pathways and/or processes for each k-means cluster and GSEA using curated KEGG pathways showing significantly enriched molecular pathways and/or processes for each k-means cluster.

**Supplementary Data File 8.** Data supporting Supplementary Figure 8 including differential gene expression analysis between Quartile1 and Quartile 2 versus Quartile 3 and Quartile 4 of the DvP axis and associated GSEA GO terms.

**Supplementary Data File 9.** Data supporting Supplementary Figure 9 and Figure 2c, including: RTN Transcription Factor seeds used to generate the RTN; RTN results; msVIPER results; VIPER Regulon Scores; VIPER Scores used to generate Supplementary Figure 9c; correlation between CIBERSORTx enrichment scores and VIPER regulon scores and the TINCR and STAU1 signature scores.

**Supplementary Data File 10.** Data supporting Figure 3 and Supplementary Figures 10 and 11, including: xCELL results; immune modulators, Pan-Immune Gene sets, immune pathway signature scores; LogR gene expression matrix comprising immunomodulatory genes; Pairwise correlation and significance between selected immunomodulatory genes; CIBERSORTx and immunomodulatory gene expression correlations; differential gene expression analysis of immune competent versus immunosuppressed sample and GSEA showing significant and differentially enriched molecular pathways and/or processes between IC and IS patient samples, CIBERSORTx and EDC gene expression correlation results; CIBERSORTx and EDC gene expression correlation results matrix; Immunomodulatory gene expression and EDC gene expression correlation results; Immunomodulatory gene expression and EDC gene expression correlation results matrix; Immune gene signature set and EDC gene expression correlation results; Immune gene signature set and EDC gene expression correlation results matrix.

**Supplementary Data File 11**. Data supporting Supplementary Figure 12 and Figure 4a and Figure 5f including; Mutational burden of whole exome sequenced samples; T cell, Macrophage and Neutrophil RNAseq Scores and Driver gene mutations; frequency tables of CN gains and losses estimated by CaSpER analysis, and frequency tables of genes gained or lost and tables of genes gained and significantly upregulated in Quartile1 and Quartile 2 samples and genes lost and significantly downregulated in Quartile 1 and 2 samples compared to Quartile 3 and 4 samples.

**Supplementary Data File 12.** Data supporting Supplementary Figure 14 including Notch, p53 and TGFβ pathway and signalling to ERK signatures.

**Supplementary Data File 13.** Data supporting Figure 4. GEMM cohort details.

**Supplementary Data File 14.** Data supporting Figures 4, 5 and Supplementary Figure 16, including: GEMM sample RNAseq manifest; Merged GEMM sample gene counts; LogR normalised gene expression values; Differentially expressed genes between GEMMs; GSEA showing significant and differentially enriched molecular pathways and/or processes between GEMMs; Selected immune gene expression and EDC gene expression correlation results; Selected immune gene expression and EDC gene expression correlation results matrix; Gene expression matrix for EDC genes; GEMM Signature genes (human orthologues); cSCC enrichment scores for GEMM phenotypes ranked by DP score; and GEMM signature gene and CIBERSORTx correlation results.

**Supplementary Data File 15.** Data supporting Figure 5e-f. HALO murine tumour immunohistochemistry immune scores.

**Supplementary Data File 16.** Data supporting Supplementary Figure 18 including total scaled murine gene expression table of murine RNaseq data from this study and Chitsazzadeh et al UV hairless mouse model[8].

**Supplementary Data File 17.** Data Supporting Supplementary Figure 19 including hair follicle cell state signature scores for our human data set.
