# Supplementary material for: Driver gene combinations dictate cutaneous squamous cell carcinoma disease continuum progression
Source: Nat Commun. 2023 Aug 25;14:5211. doi: 10.1038/s41467-023-40822-9 (PMC10457401; doi:10.1038/s41467-023-40822-9)
Supplement: Supplementary file 21 — Reporting Summary [file 41467_2023_40822_MOESM21_ESM.pdf]

nature portfolio

Corresponding author(s): Gareth Inman

Last updated by author(s): 2023-7-10

Reporting Summary

Nature Portfolio wishes to improve the reproducibility of the work that we publish. This form provides structure for consistency and transparency in reporting. For further information on Nature Portfolio policies, see our Editorial Policies and the Editorial Policy Checklist.

Statistics

For all statistical analyses, confirm that the following items are present in the figure legend, table legend, main text, or Methods section.

n/a	Confirmed	
☐	☒	The exact sample size (n) for each experimental group/condition, given as a discrete number and unit of measurement
☐	☒	A statement on whether measurements were taken from distinct samples or whether the same sample was measured repeatedly
☐	☒	The statistical test(s) used AND whether they are one- or two-sided<br>*Only common tests should be described solely by name; describe more complex techniques in the Methods section.*
☒	☐	A description of all covariates tested
☐	☒	A description of any assumptions or corrections, such as tests of normality and adjustment for multiple comparisons
☐	☒	A full description of the statistical parameters including central tendency (e.g. means) or other basic estimates (e.g. regression coefficient) AND variation (e.g. standard deviation) or associated estimates of uncertainty (e.g. confidence intervals)
☐	☒	For null hypothesis testing, the test statistic (e.g. F, t, r) with confidence intervals, effect sizes, degrees of freedom and P value noted<br>*Give P values as exact values whenever suitable.*
☒	☐	For Bayesian analysis, information on the choice of priors and Markov chain Monte Carlo settings
☒	☐	For hierarchical and complex designs, identification of the appropriate level for tests and full reporting of outcomes
☐	☒	Estimates of effect sizes (e.g. Cohen's d, Pearson's r), indicating how they were calculated

Our web collection on statistics for biologists contains articles on many of the points above.

Software and code

Policy information about availability of computer code

Data collection	For Image analysis Indica labs HALO® (Indica labs , v3.1.1076.363) software was used.
Data analysis	RNA sequencing raw read data was analysed using the nf-core/rnaseq pipeline. Sequencing reads were mapped to human genome assembly GRCh38 or mouse genome assembly GRCm38 using STAR aligner. The following R packages were used for transcriptional data analyses DESeq2, ggpubr, bbplot, ggplot2, ComplexHeatmap, circlize, GViz2, corrplot, FactoMineR, factoextra, Rtsne, GSVA, genefu, cluster, ConsensusClusterPlus, RTN, msviper, RedeR, xCELL, CaSpER, msigdbr and UMAP. Data analysis of murine tumour development and tumour IHC were undertaken in Graphpad Prism version 9.

For manuscripts utilizing custom algorithms or software that are central to the research but not yet described in published literature, software must be made available to editors and reviewers. We strongly encourage code deposition in a community repository (e.g. GitHub). See the Nature Portfolio guidelines for submitting code & software for further information.

Data

Policy information about availability of data

All manuscripts must include a data availability statement. This statement should provide the following information, where applicable:

- Accession codes, unique identifiers, or web links for publicly available datasets
- A description of any restrictions on data availability
- For clinical datasets or third party data, please ensure that the statement adheres to our policy

All data supporting the findings of this study are available within the Article and the Supplementary Information. Murine transcriptomic data are available through the Gene Expression Omnibus (GEO) with the accession code GSE199070 and human transcriptomic datasets are available through the open access NCBI BioProject (ID PRNJA844527)

Human research participants

Policy information about studies involving human research participants and Sex and Gender in Research.

Reporting on sex and gender	The sex of patients from which biological samples were collected is reported in our clinical manifest in the supplementary data file 1 accompanying the manuscript.
Population characteristics	The age of patients at diagnosis is reported in our clinical manifest in the supplementary data file 1 accompanying the manuscript.
Recruitment	The samples analyzed in this study were assembled retrospectively from samples obtained from consented patients and stored at participating sites
Ethics oversight	This study was approved by the East of Scotland Research Ethics Service (REC reference 08/S1401/69), The Ethics and Scientific Committee of A. Sygros Hospital (Ref 2353/3-11-2016) and The University of California, San Francisco Institutional Review Board and was conducted according to the Declaration of Helsinki Principles. All patients participating in the study provided written, informed consent.

Note that full information on the approval of the study protocol must also be provided in the manuscript.

Field-specific reporting

Please select the one below that is the best fit for your research. If you are not sure, read the appropriate sections before making your selection.

☒ Life sciences ☐ Behavioural & social sciences ☐ Ecological, evolutionary & environmental sciences

For a reference copy of the document with all sections, see nature.com/documents/nr-reporting-summary-flat.pdf

Life sciences study design

All studies must disclose on these points even when the disclosure is negative.

Sample size	For all in-vivo experiments, power analyses were carried out to determine cohort sizes based upon effect size and SD derived from unpublished experiments in similar genetic models previously carried out within the lab, and from early pilot studies which were carried pout within experimental and control cohorts. Power analyses were carried out using the G*power software package 3.1.9.4 (HHU Dusseldorf), typically defining alpha=0.05 and beta=0.2. For animal studies this also respected the limited use of animals in line with 3R system: replacement, Reduction, Refinement.
Data exclusions	No data were excluded.
Replication	For IHC analysis of murine tumours tissue sections from at least six tumours from each genotpye each harvested from a different individual were stained and all samples were included in the analyses.
Randomization	To minimise genetic variability all experimental and control animals were generated from individual breeding colonies. Control and experimental animals were co-housed independent of genotype and cohorts comprised abalance of both male and female animals. Randomisation of the human samples was not performed as all samples passing RNAseq QC metrics were included in this study.
Blinding	Researchers were not blinded to murine genotypes during the study and data collection for animal welfare reasons which included implementation of correct monitoring frequencies and processes. The investigators were blinded to genotype during processing of RNAseq samples and data analysis of H&E and IHC.

Reporting for specific materials, systems and methods

We require information from authors about some types of materials, experimental systems and methods used in many studies. Here, indicate whether each material, system or method listed is relevant to your study. If you are not sure if a list item applies to your research, read the appropriate section before selecting a response.

Materials & experimental systems

n/a	Involved in the study
	☒ Antibodies
☒	☐ Eukaryotic cell lines
☒	☐ Palaeontology and archaeology
	☒ Animals and other organisms
☒	☐ Clinical data
☒	☐ Dual use research of concern

Methods

n/a	Involved in the study
☒	☐ ChIP-seq
☒	☐ Flow cytometry
☒	☐ MRI-based neuroimaging

Antibodies

Antibodies used	CD3 (1:100, Abcam, # ab16669), CD4 (1:500, eBioscience, #14-9766-82), CD8 (1:500, eBioscience, #14-0808-82), F4/80 (1:100, Abcam, #ab6640) and LY6G (1:60000, BioXcell, #BE0075-1). Secondary antibodies used were Rabbit EnVision solution (Agilent, #K5007) for CD3 and Rat ImmPRESS solution (Vector laboratories MP744415) for CD4, CD8, F4/80 and LY6G.
Validation	1. CD3 (1:100, Abcam, # ab16669, clone SP7), validated for Flow Cyt, IHC-P, WB, mIHC, Reacts with: Mouse, Rat, Human<br>2) CD4 (1:500, eBioscience, #14-9766-82, clone 4SM95), validated for WB, IHC, IF. Species reactivity Mouse<br>3) CD8 (1:500, eBioscience, #14-0808-82, clone 4SM15), validated for WB, IHC, IF, Flow cytometry. Species reactivity Mouse<br>4) F4/80 (1:100, Abcam, #ab6640, clone A3-1), validated for ICC, IF, Flow cytometry. Species recativity: Mouse<br>5) LY6G (1:60000, BioXcell, #BE0075-1, clone RB6-8C5) validated for IF, IHC, Flow cytometry and in vivo depletion of nuetrophils and MDSC. Species reactivity Mouse

Animals and other research organisms

Policy information about studies involving animals; ARRIVE guidelines recommended for reporting animal research, and Sex and Gender in Research

Laboratory animals	Mice of both sexes from 2-6 months of C57BL6/J, S129 background were used.
Wild animals	no wild animals were used in this study
Reporting on sex	data reported is aggregated data from both sexes of mice and the sex of each individual mouse in the study is found in the Supporting data tables
Field-collected samples	no field collected samples were used in this study
Ethics oversight	All animal experiments were performed in accordance with UK Home Office regulations (project licence 70/8646), and adherence to the ARRIVE guidelines, and were reviewed and approved by the Animal Welfare and Ethical Review Board of the University of Glasgow

Note that full information on the approval of the study protocol must also be provided in the manuscript.
